# The Role of Vacuum Assisted Closure in Patients with Pressure Ulcer and Spinal Cord Injury: A Systematic Review

**DOI:** 10.29252/wjps.8.3.279

**Published:** 2019-09

**Authors:** Avraam Ploumis, George Mpourazanis, Christina Martzivanou, Pantelis Mpourazanis, Areti Theodorou

**Affiliations:** 1 Department of Medicine, University of Ioannina, Ioannina, Greece;; 2 Department of Chemistry, University of Ioannina, Ioannina, Greece

**Keywords:** Pressure ulcer, Spinal cord injury, Vacuum assisted closure, Intermittent negative pressure

## Abstract

**BACKGROUND:**

Patients with spinal cord injury (SCI) tend to develop pressure ulcers (PrUs) because of prolonged immobility. This study assessed the efficacy of vacuum assisted closure for healing of PrUs in individuals with SCI.

**METHODS:**

In a systematic review, CINAHL, PubMed, Cochrane Clinical Trials, DARE, MEDLINE, Scopus, Embase, Science Direct, PsycInfo and Spinal Cord Journal were searched in March 2019. The search combined related terms for pressure ulcer, spinal cord injury, and vacuum assisted closure. Each database was searched from its inception with no restrictions on year of publication.

**RESULTS:**

The search yielded 7 studies for inclusion in a qualitative analysis. The studies included a variety of methodologies, specifically 2 randomized controlled trials, 2 assessor-blinded crossover and retrospective cohort study, 1 prospective non-randomized trial, 1 randomized case study and 1 case report. The meta-analysis was unsuccessful. Only descriptive results mean±SD were reported as well as time to heal and time to discharge after admission.

**CONCLUSION:**

The studies that we included in our qualitative synthesis showed that vacuum assisted closure promoted the healing of PrUs in individuals with SCI.

## INTRODUCTION

Pressure ulcers are localized areas of tissue necrosis that tend to develop, when soft tissue is compressed between a bony prominence and an external surface for a prolonged period of time. Patients with spinal cord injury (SCI) tend to develop pressure ulcers (PrUs) because of prolonged immobility. 41% of patients with SCI in their first year present pressure ulcers.^1^^,^^2^ Moreover, 37% of SCI patients develop pressure ulcers during their acute care hospitalization.^3^ Factors contributing to PrUs are immobility, moisture and irritation to the skin.^4^

Management of PrUs includes skin checks, proper seating systems, offloading pressure-prone areas by positioning, and healthy nutrition and surgery. Surgical restoration uses flap techniques such as fasciocutaneous or myocutaneous flaps. Conventional dressing therapy after the initial surgical debridement consists of moist gauzes saturated with topical agents like sodium hypochlorite. Furthermore, foams, hydrocolloids, hydrogels and transparent film dressing are used.^5^

Vacuum-assisted closure (VAC) is a system that promotes the healing of wounds. VAC is based on the principal that negative pressure applied to the wound will promote an improved environment for wound healing.^6^ The VAC system applies topical negative pressure to the wound using a polyurethane reticulated open-cell foam dressing, or a polyvinyl alcohol foam dressing. When VAC is applied to the area under negative pressure conditions, the tissue promotes cell mitosis and proliferation of reparative granulation tissue.^7^ In this research, data from the scientific literature on PrUs in patients with SCI were presented. The aim of this work was to review the role of VAC in PrUs of SCI patients.

## MATERIALS AND METHODS

The study has been designed and the results have been reported based on the PRISMA statement (see Supplementary Checklist).^8^ This review included prospective non-randomized trial, randomized, assessor-blinded crossover pilot, retrospective observational cohort study, randomized controlled trial and case study involving patients with PrUs and SCI, and the treatments. We searched the following databases: CINAHL, PubMed, Cochrane EBM Reviews, Cochrane Clinical Trials, DARE, MEDLINE, Scopus, Embase, Science Direct, PsycInfo and Spinal Cord Journal.

Search terms were “PrUs” AND “VAC” OR “SCI”. These studies excluded: (i) studies not providing data on baseline score or end-point outcome, and (ii) studies providing only qualitative data. Data extraction was undertaken separately for each intervention. All relevant information was extracted for each study: first author, year of publication, type of study, study duration, age of patients, location of ulcer, number of patients, types of therapy and complications. The search was limited to English language articles. The reference lists of the included articles have also been searched for additional studies. 

## RESULTS

**Fig. 1:**
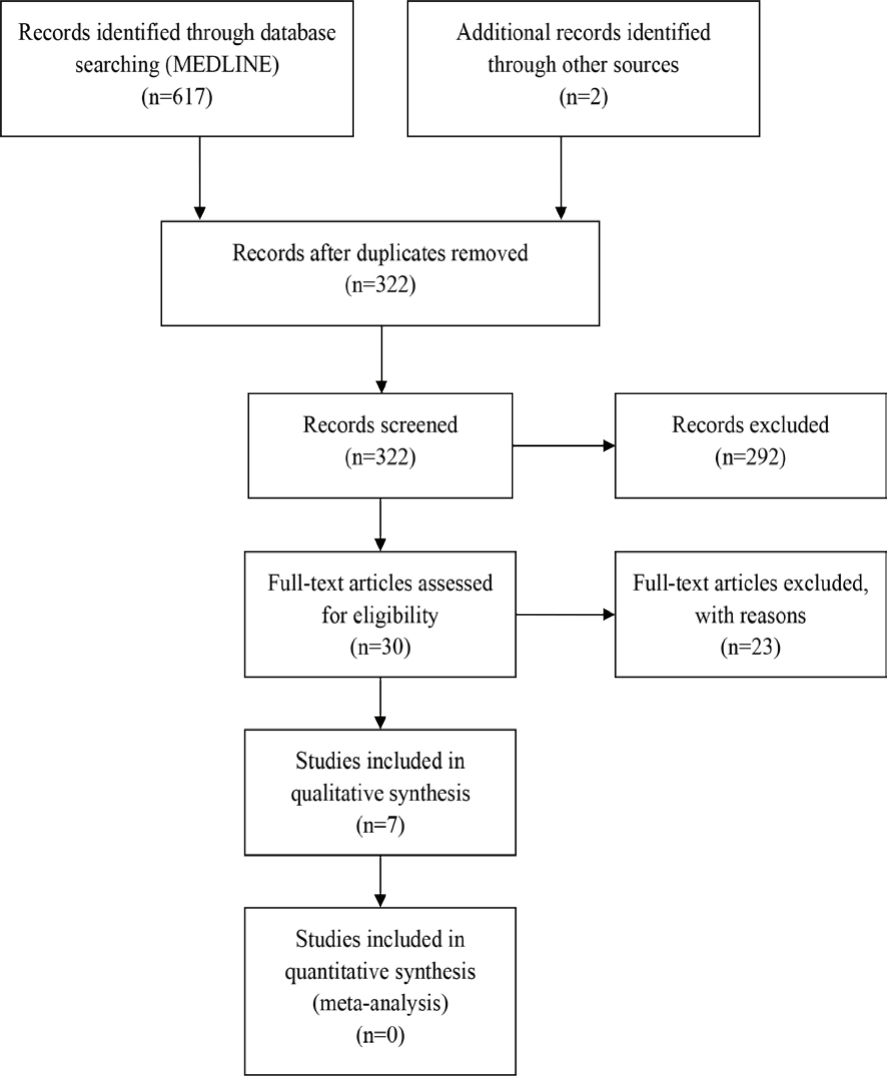
PRISMA 2009 flow diagram

Figure 1 shows the search algorithm. An overview of trial characteristics is provided in Table 1, where most of the patients had pressure ulcers in the ischial tuberosities, stage 3 and 4. The total patient/participant’s population was 224, the range of age was 16-87 years (mean: 48.3) and the therapies included multiple variations of VAC, such as topical negative pressure/device (TNP/TND) and negative pressure wound therapy (NPWT). 

**Table 1:** An overview of trial characteristics

**Author/ Country**	**Year**	**Type of study**	**Study duration**	**Age (mean or median)**	**Location of ulcer**	**Number of patients**	**Types of therapy**	**Results**	**Complications**
Papp A. Anthony Canada	2018 December	Prospective non-randomized trial	2 years	55.8 (24-84) years	Mostly over the ischial tuberosities followed by sacrum and greater trochanters	37	iNPWT	Decrease in complications and number of open recurrent wounds at 3 months after discharge from hospital. Reduced length of stay in hospital from iNPWT therapy.	2 patients had hematoma and one of them also had wound edge necrosis.
Sundby Øyvind Heiberg Norway	2018 April	Randomized, assessor-blinded crossover pilot study	16 weeks	57	Feet	4	INP device	Both photographic wound assessment tool (PWAT) and wound surface area (WSA) were improved in the group where intermittent negative pressure (INP) as well standard wound care (SWC) were used, in comparison to SWC-treatment alone.	1 patient withdrew due to bleeding from the ulcer, after 1 session of INP.
Kreutzträger Martin Germany	2018 January	Retrospective observational cohort study	3 years	51 (18-87 years)	Ischium, trochanter major, and sacral regions (4^th^ grade)	36	NPWT	Negative pressure wound treatment (NPWT) has similar complications rates and hospital length of stay with the wound irrigation and dressings with Lavanid. Nevertheless, the time of mobilization in a wheelchair was longer in the group with NPWT.	Pneumonia* (is referred to all subjects and not SCI patients only).
Dwivedi Mohan Krishna India	2016 April	Randomized controlled trial (RCT)	9 weeks	38.38 ± 7.65 (16-60) years	Sacral pressure ulcers stage 3 and 4.	21	NPWT with NPD	Negative pressure device (NPD) is better than standard wound care procedures and cost-effective for management of PU (47% less cost).	No complications were referred.
De Laat Erik Hew Netherlands	2011 December	Randomized controlled trial (RCT)	6 weeks	48,7 years	Not referred	6	TNP with VAC	Topical negative pressure (TNP) resulted in almost 2 times faster wound healing than treatment with sodium hypochlorite and is safe to use in patients with difficult-to-heal wounds.	1 patient with small arterial bleed and another 1 had a wound with bacterial growth.
Citak Mustafa Germany	2010 March	Case report	3 months	43	Ischium (4^th^ grade)	1	VAC	The VAC technique is a safe, easy, and effective means in chronic wound care management, however inadequate with deep ulcer wounds with underlying osteomyelitis.	Superinfection, necrotizing fasciitis and further progress of the osteomyelitis of the patient.
Coggrave Maureen England	2002 March	Case study (7 cases)	2 years	44,4 (30-72) years	Trochanter, sacrum and ischium	7	TNP	Decrease in wound dimensions and wound volumes. Reduction in local edema and nursing time spent on dressings.	2 patients developed rashes or excoriation.

Incisional negative pressure wound therapy (iNPWT), Intermittent negative pressure (INP), Negative pressure wound therapy (NPWT), negative pressure device (NPD), Vacuum assisted closure (VAC), Topical negative pressure (TNP)

The healing time differed in our studies, from 1 month to almost 2 years. A homogenous result was found in the changes of VAC dressings that were done approximately once to thrice a week, which was demonstrated in Table 2. The time of discharge of the patients after admission was only referred in 2 articles, where both of them had a similar outcome of 3 months. Table 2 refers to the articles which included information about either time to heal, changes of VAC dressings or time of discharge after admission.

**Table 2:** The articles which included information about either time to heal, changes of Vacuum assisted closure (VAC) dressings or time of discharge after admission

**Author**	**Time to heal**	**Changes of VAC dressings**	**Time of discharge after admission**
Papp	24,7 months	Every 7 days	-
Kreutzträger	Mean: 34 days	Every 3 days	Mean: 100 days
Dwivedi	6 weeks	Every 7 days	-
De Laat	6 weeks until 50% of the wound volume reduction was reached	3 times a week	-
Citak	-	Every 5 days	3 months
Coggrave	Mean: 35,7 days	Every 4-7 days	-

## DISCUSSION

The purpose of our study was to review the role of VAC in SCI-patients with PrUs. Seven articles that fulfilled this purpose were found. VAC was more commonly used in ischial tuberosities ulcers needing changes up to 3 times per week and time to discharge approximately 3 months. According to clinical and laboratory experience, the VAC system helped removing interstitial fluid from wounds, decreasing bacterial colonization and increasing wound vascularity. 

VAC can be used in a variety of diseases such as chronic open wounds which appear and are a common problem in patients with spinal cord injury, in postoperative spinal surgical infections and in diabetic ulcers.^9^ It is safe to use for the management of infected wounds, open fracture wounds and related soft tissues complications and acute soft tissue wounds.^10^ Although most of the patients had PrUs near the ischium, they were cases where patients had pressure ulcers on the feet and on the rather difficult to heal area of the sacrum.^11^

Nonetheless, the results in all studies/cases were satisfying. Furthermore, VAC devices were indicated for serious traumatic and dehisced wounds. In addition, VAC used in posttraumatic injuries as a therapy of infections had also been referred to, because of its great antimicrobial agents. Lastly, VAC therapy used in pressure ulcers not only in SCI patients, but patients in general lead to a considerable decrease in costs, due to a cutback in resource utilization, like inpatient care, antibacterial agents and outpatient treatment visits.^11^

We found that by using VAC treatment patients had indeed shorter curing time than those who were using regular therapies as the median treatment time with VAC was shown to be shorter than the corresponding time in the group with the traditional medication. In addition, SCI patients who received negative pressure as therapy showed faster reduction in size and depth of the ulcers as well as faster granulation tissue formation in contrast to the SCI patients with standard care. 

The changes of the dressings were also different, for example in the treatment group with conventional dressings, changes had to be done 2-3 times a day versus the changes in the VAC-treatment group which were approximately 1-3 times a week.^12^ Last, a greater decrease was noted in in-hospital complications, the average length of stay and finally the number of open wounds at a 3 months’ follow up in the group cured by VAC treatment than of that with no VAC therapy.^13^

Several limitations were needed to be considered, when interpreting the results of this study. First of all, the studies themselves were being non-randomized and small in the patients’ number, thus having a small sample size. Secondly, the unsuccessful meta-analysis of the complete study itself has to be noted. Another point which has to be considered is the withdrawal of some patients from the trials, due to complications caused by the use of VAC. Lastly, the VAC therapy shows a great limitation in its use in ulcers near the low sacrum, where it cannot be easily applied.^12^

Despite the findings of our research that support the use of VAC, further studies which include more randomized trials will be needed in order to fully support and determine the clinical and cost-effectiveness of VAC therapy in comparison with other treatments. The studies we included in our qualitative synthesis show that VAC improves the curing of PrUs in individuals with SCI and reduces the overall curing time and stay-in-hospital length as well as in-hospital complications.^13^ The VAC technique is secure for home use, simple to apply and is competent in chronic wounds in patients not only with SCI, but also in sufferers with pressure ulcer, caused by diabetes, infections and post-traumatic injuries.

## CONFLICT OF INTEREST

The author(s) declares no potential conflicts of interest with respect to the research, authorship and/or publication of this article. The author(s) received no financial support for the research, authorship and/or publication of this article.
